# New Uses for Old Drugs: The Tale of Artemisinin Derivatives in the Elimination of Schistosomiasis Japonica in China

**DOI:** 10.3390/molecules190915058

**Published:** 2014-09-19

**Authors:** Yi-Xin Liu, Wei Wu, Yue-Jin Liang, Zu-Liang Jie, Hui Wang, Wei Wang, Yi-Xin Huang

**Affiliations:** 1 Jiangsu Institute of Parasitic Diseases, 117 Yangxiang, Meiyuan, Wuxi 214064, China; E-Mails: yixinliu@sina.com.cn (Y.-X.L.); wuwei8858@aliyun.com (W.W.); 2 Key Laboratory on Technology for Parasitic Disease Prevention and Control, National Health and Family Planning Commission, 117 Yangxiang, Meiyuan, Wuxi 214064, China; 3 Jiangsu Provincial Key Laboratory of Molecular Biology of Parasites, 117 Yangxiang, Meiyuan, Wuxi 214064, China; 4 Department of Microbiology and Immunology, The University of Texas Medical Branch, 301 University Blvd, Galveston, TX 77555, USA; E-Mails: yu2liang@utmb.edu (Y.-J.L.); zujie@utmb.edu (Z.-L.J.); 5 Department of Integrative Biology and Pharmacology, The University of Texas Health Science Center at Houston, 6431 Fannin St, Houston, TX 77030, USA; E-Mail: hui.wang@uth.tmc.edu

**Keywords:** schistosomiasis japonica, artemisinin, artemether, artesunate, dihydroartemisinin, elimination, China

## Abstract

Artemisinin (qinghaosu), extracted from the Chinese herb *Artemisia annua* L. in 1972, and its three major derivatives—artemether, artesunate and dihydroartemisinin—were firstly identified as antimalarials and found active against all species of the malaria parasite. Since the early 1980s, artemisinin and its derivatives have been found efficacious against Schistosoma spp., notably larval parasites, and artemisinin derivatives have played a critical role in the prevention and treatment of human schistosomiasis in China. Currently, China is moving towards the progress of schistosomiasis elimination. However, the potential development of praziquantel resistance may pose a great threat to the progress of elimination of schistosomiasis japonica in China. Fortunately, these three major artemisinin derivatives also exhibit actions against adult parasites, and reduced sensitivity to artemether, artesunate and dihydroartemisinin has been detected in praziquantel-resistant *S. japonicum*. In this review, we describe the application of artemisinin derivatives in the prevention and treatment of schistosomiasis japonica in China, so as to provide tools for the global agenda of schistosomiasis elimination. In addition to antimalarial and antischistosomal actions, they also show activities against other parasites and multiple cancers. Artemisinin derivatives, as old drugs identified firstly as antimalarials, continue to create new stories.

## 1. Introduction

Schistosomiasis, caused by the infection with the blood flukes of the genus Schistosoma, is a major neglected parasitic disease in the tropical and subtropical regions [1]. It is estimated that over 200 million people are infected with the parasite, with a further 800 million at risk of infection [2]. Schistosomiasis japonica, caused by the infection with the parasite *S. japonicum*, is endemic in the People’s Republic of China, Philippines, and Indonesia [3]. In China, this parasitic disease remains a major public health concern and is one of the four priorities in communicable disease control defined by the central government [4]. Although great success has been achieved [5,6,7], the current endemic foci are mainly concentrated on the marshland and lake regions of five provinces (Hunan, Hubei, Jiangxi, Anhui and Jiangsu) along the middle and lower reaches of the Yangtze River and some mountainous regions of two provinces of Yunnan and Sichuan, with over 0.8 million people infected [8].

Since the introduction in 1970s, praziquantel has replaced other agents to become the only drug of choice for the treatment of human schistosomiases, due to its high efficacy, easy administration, few side effects and low cost [9,10,11]. Following the repeated, extensive application, there is a concern however about the emergence of praziquantel-resistant schistosome populations [12,13,14]. In laboratory, praziquantel resistance has been successfully induced in *S. mansoni* strains following treatment with subcurative doses [15,16], and drug-resistant isolates have been isolated from the endemic foci in Africa [17,18]. In addition, there are many *S. haematobium*-infected cases with failure in parasite clearance following standard treatments [19,20,21]. In China, praziquantel is currently the only market-available chemical used for the treatment of *S. japonicum* infections, and praziquantel-based chemotherapy, a major part of the national schistosomiasis control program, has been implemented to control the morbidity and reduce the prevalence and intensity of *S. japonicum* infection for more than 30 years [22,23,24]. Although no evidence of resistance to praziquantel has been detected yet in field isolates of *S. japonicum* [25,26,27], the emergence of drug resistance has been experimentally induced in the laboratory, proving that *S. japonicum* may develop resistance to praziquantel under drug selection pressure [28,29]. The potential development of praziquantel resistance would challenge the elimination of schistosomiasis japonica in China, since praziquantel-based chemotherapy remains highly effective for controlling the morbidity and reducing the prevalence [30]. Screening and development of novel antischistosomal agents, as alternatives to praziquantel, are therefore urgently needed [31].

In 1972, Chinese scientists extracted artemisinin (qinghaosu) from the Chinese herb *Artemisia annua* L, and the compound has been found efficacious against all species of the malaria parasite, even chloroquine-resistant *Plasmodium falciparum* [32,33]. The encouraging results led to the development of multiple artemisinin derivatives, including artemether, artesunate and dihydroartemisinin, which were found to possess higher anti-malarial activities than artemisinin [34,35,36,37]. Currently, artemisinin derivatives are widely used for the treatment of malaria worldwide [37,38]. Since the early 1980s, artemisinin and its derivatives have been found to be active against Schistosoma spp., notably larval stages, and artemisinin derivatives have played a critical role in the prevention and treatment of human *S. japonicum* infections in China [39,40,41,42,43]. Since praziquantel is primarily active against adult stages and relatively ineffective against juvenile worms [44], the combination of praziquantel and artemisinin derivatives may cover all developmental stages of the parasite, and the combination therapy may overcome the problem of drug resistance [45]. In this review, we focus on the role of three major artemisinin derivatives, artemether, artesunate and dihydroartemisinin, in the elimination of schistosomiasis japonica in China.

## 2. Artemether

### 2.1. Artemether Monotherapy

Artemether is a methyl ether derivative of artemisinin. Its antischistosomal action was first described in 1980, and the agent is active against *S. japonicum*, especially on 5–21-day-old juvenile worms [46]. The exciting results from the subsequent studies in experimental animal models indicated the need for the clinical trials in the major endemic foci of China [47,48,49]. Table 1 summarizes the major clinical trials reported with aims to test the efficacy of artemether against human *S. japonicum* infections. Multiple doses of artemether at 6 mg/kg over 15-d intervals were found to achieve 60.8% to 100% protection for the prevention of *S. japonicum* infection, and increased doses resulted in improved efficacies; following artemether therapy, no acute case with schistosomiasis occurred, and only mild, transient treatment-associated adverse events were observed [50,51,52,53,54,55,56,57]. Meta analyses of these clinical trials confirmed that repeated dosing of artemether significantly reduces the incidence of *S. japonicum* infections as compared with the placebo, and it is more effective to use multiple doses of artemether with 2-week intervals for prevention against *S. japonicum* infection [43,45].

### 2.2. Artemether-Praziquantel Combination

Since praziquantel and artemether act against different developmental stages of *S. japonicum*: praziquantel against adult worms and artemether against schistosomula, a combination therapy may cover all developmental stages and result in an improved antischistosomal efficacy. To test the hypothesis, animal experiments were performed to evaluate the efficacy of praziquantel-artemether combination against *S. japonicum* infection.

**Table 1:** Clinical trials reported to test the preventive efficacy of artemether against *S. japonicum* infection.

Treatment Regimen	Preventive Efficacy (%)	Endemic Type of the Pilot	Adverse Events	Reference
Four doses of artemether (6 mg/kg) given once every 15 days	60.8	Islet with embankment	None	[50]
Four doses of artemether (6 mg/kg) given once every 15 days	71.7	Mountainous region	6 cases developed mild, transient dizziness, nausea, abdominal pain and diarrhea after the first treatment	[51]
Ten doses of artemether (6 mg/kg) given once every 15 days	75.9	Mountainous region	Mild, transient dizziness (0.67%), burning sensation in the upper abdomen (0.45%), abdominal pain (0.38%), fever (0.18%), abdominal distension (0.11%), vomiting (0.41%), abdominal pain (0.23%), diarrhea (0.13%), and palpitation (0.07%)	[52]
Nine doses of artemether (6 mg/kg) given once every 15 days	94	Islet with embankment	None	[53]
Ten doses of artemether (6 mg/kg) given once every 15 days	100	Marshland region	None	[54]
Two doses of artemether (6 mg/kg) given 15 days apart, with an additional dose 15 days post-exposure	91	Marshland region	None	[55]
Nine to eleven doses of artemether (6 mg/kg) given once every two weeks	94.6	Lake region	None	[56]
Eleven doses of artemether (6 mg/kg) given once every 15 days	94.47	Marshland region	Transient dizziness (0.65%), burning sensation in the upper abdomen (0.45%), abdominal pain (0.38%), fever (0.18%), abdominal distension (0.11%) and vomiting (0.09%),	[57]

In rabbits infected with 7–14-day-old schistosomula and 42-day-old adult schistosomes, the combined treatment of praziquantel and artemether reduced total worm burdens by 79%–92% and female worm burdens by 80%–93%, which were significantly greater than those caused by treatment with 50 mg/kg praziquantel (28%–66% total worm burden reduction and 26%–65% female worm burden reduction) or 15 mg/kg artemether alone (44%–56% total worm burden reduction and 35%–54% female worm burden reduction), using the same dosages and schedules [58]. A further study in rabbits experimentally infected with 42- or 56-day-old adult *S. japonicum*, praziquantel-artemether combination achieved total worm burden reductions of 96%–99% and female worm burden reductions of 99%–100%, while treatment with praziquantel administered at a single dose of 40 mg/kg alone resulted in total worm reduction rates of 87%, and female worm reduction rates of 88%–96%, and a single dose of 15 mg/kg artemether resulted in total worm reduction rates of 25%–33% and female worm reduction rates of 12%–31% [59]. It is therefore considered that a praziquantel-artemether combination at lower doses is safe and more effective than administration with praziquantel or artemether alone.

The exciting findings from animal experiments provided a foundation for clinical trials in humans. A randomized, double-blind, placebo-controlled trial in the Dongting Lake region of China between May 2003 and December 2005 showed that treatment with 60 mg/kg praziquantel combined with 6 mg/kg artemether, 60 mg/kg praziquantel combined with artemether placebo, 120 mg/kg praziquantel combined with 6 mg/kg artemether, and 120 mg/kg praziquantel combined with artemether placebo achieved treatment efficacies of 98%, 96.4%, 97.7%, and 95.7% in the treatment of acute schistosomiasis japonica, respectively, and the combination of artemether and praziquantel chemotherapy did not improve the treatment efficacy compared with praziquantel alone [60]. In addition, administration of artemether caused minor adverse events in 6.3% of the subjects within 4 h of the treatment, including allergy, nausea, vomiting and abdominal discomfort, while 26.3% suffered pain in the upper abdominal region after the first or second praziquantel treatment [60]. Due to the inconsistency between animal experiments and clinical trials, further studies are required to validate the human findings before the policy-making on the management of schistosomiasis japonica in China.

### 2.3. Recommended Treatment Regimen

At present, artemether is mainly used for the prevention of schistosome infections, and the following regimen is recommended: artemether is administered at a dose of 6 mg/kg starting one or two weeks after contact with infested water during the transmission season, followed repeated treatment every 1–2 weeks for several doses, with an additional treatment given 15 days post-exposure. However, the treatment is contraindicated in subjects with early pregnancy, severe liver, renal or hematologic diseases, or allergy to artemether. In addition, oral administration of artemether at a dose of 6 mg/kg daily for 5–7 days may be given in cases with allergy to praziquantel, since the agent is also active against adult worm of the parasite [61].

## 3. Artesunate

### 3.1. Artesunate Monotherapy

The antischistosomal action of artesunate was firstly described in 1980 [46]. To assess the prophylactic effect of artesunate against *S. japonicum*, artesunate was given to experimentally infected mice, rabbits and dogs at doses of 300, 20–40 and 30 mg/kg 7 days post-infection once a week for 4–6 weeks, and the treatments resulted in worm burden reductions of 77.5%–90.66%, 99.53% and 97.10%, respectively [62,63]. In addition, treatment with artesunate at a dose of 16 mg/kg given once a week for 4 weeks starting 7 days post-infection was found to effectively prevent the development of acute schistosomiasis and suppress egg production in rabbits [64], and artesunate was found active against various developmental stages of *S. japonicum* in experimentally infected mice, notably 7-day schistosomulum [65].

The exciting animal findings encouraged human trials to evaluate the efficacy of artesunate against *S. japonicum* infections. Table 2 summarizes the major clinical trials conducted to test the preventive activity of artesunate against the parasite. These clinical trials demonstrated that multiple doses of artesunate at 6 mg/kg given once a week at 7 days apart achieved 68.19%–100% protection against human *S. japonicum* infection and effectively prevented the development of acute cases of schistosomiasis japonica [66,67,68,69,70,71,72,73,74,75]. A recent study reported a reduced sensitivity of artesunate against *S. japonicum* after 10 years of application in China [76]; however, further trials are required to validate the finding that *S. japonicum* shows reduced susceptibility to artesunate [77].

### 3.2. Artesunate-Praziquantel Combination Therapy

Since artesunate and praziquantel are two antischistosomal agents targeting schistosomula and adults, respectively, the combined treatment was hypothesized to increase the efficacy. To test the hypothesis, experimental studies were performed in mice and rabbits. Co-administration of artesunate and praziquantel, or administration of praziquantel, followed by artesunate, was found to significantly reduce the activities of the artesunate against *S. japonicum* schistosomula, and administration of artesunate first, followed by praziquantel, resulted in similar antischistosomal activities to those observed with artesunate alone, while praziquantel-artesunate combination treatment significantly reduced the activities of praziquantel against adult worms [78]. However, the administration of artesunate first followed by praziquantel was found to achieve a comparative antischistosomal activity to that caused by treatment with artesunate alone in experimentally infected rabbits [79,80].

To confirm the results from animal experiments, clinical trials were designed and performed during the schistosomiasis transmission season. In the major flood of 1998, the administration of artesunate starting within 7 days of contact with infested water, at an oral dose of 300 mg given 7 days apart for 4 times, and treatment with praziquantel at a single dose of 1.2 g within 23–25 days of contact with the infested water, with an additional dosing 7 days post-exposure, significantly improved the protective efficacy compared with the controls (1.62% *vs.* 14.29%, *p* < 0.01), and the treatment-associated adverse events included mild gastrointestinal reaction in 6 cases and transient premature beats in three cases [81].

**Table 2:** Clinical trials conducted to test the preventive efficacy of artesunate against *S. japonicum* infection.

Treatment Regimen	Preventive Efficacy (%)	Endemic Type of the Pilot	Adverse Events	Reference
Eight doses of artesunate (6 mg/kg) given once a week starting 7 days after contact with infested water	100	Islet with embankment	Dizziness (1.71%), abdominal pain (1.44%), headache (1.16%), diarrhea (0.51%), weakness (0.47%), nausea (0.11%)	[66]
Ten doses of artesunate (6 mg/kg) given once every 15 days	96.8	Marshland region	Nausea (4.64%), dizziness (4.33%), weakness (3.4%), abdominal distention (0.62%), fever (0.62%) and palpitation (0.62%), abdominal pain (0.31%) and chest tightness (0.31%)	[67]
Eight doses of artesunate (6 mg/kg) given once every a week	100	Islet with embankment	Dizziness (2.77%), headache (1.47%), abdominal pain (1.9%), and weakness (0.97%)	[68]
Eight doses of artesunate (6 mg/kg) given once every 15 days	100	Islet with embankment	0.4% incidence of side effects including dizziness, weakness, diarrhea, abdominal pain and reduced appetite	[69]
Four doses of artesunate (6 mg/kg) given once every 15 days	80.94	Islet with embankment	Dizziness (8.79%), weakness (2.2%), abdominal pain (1.46%), shivering (1.46%), vomiting (1.1%), nausea (1.1%), fever (0.73%)	[70]
Four doses of artesunate (6 mg/kg) given once every 15 days	100	Marshland and lake region	Dizziness (2.8%), abdominal distention (1.87%) and vomiting (0.93%)	[70]
Three doses (300 mg) of artesuante 7 to 10 days after contact with infested water, with an addition 300 mg post-exposure	100	Lake region	Unreported	[71]
Six doses of artesunate (6 mg/kg) given once every 15 days	68.19	Islet with embankment	Dizziness (5.3%), weakness (3.97%), nausea (2.65%), abdominal pain (1.99%), fever (1.32%)	[72]
Twelve doses of artesuante (6 mg/kg) given once every 15 days, with an additional dose 15 days post-exposure	100	Lake region	6 cases with dizziness and nausea, 1 case with fever, and 1 case with gastrointestinal discomfort	[73]
Three doses of artesunate (6 mg/kg) given once every 15 days	100	Islet with embankment	Mild dizziness, headache, weakness, gastrointestinal discomfort	[74]
Five doses of artesunate (6 mg/kg) given once every 15 days	100	Islet with embankment	Mild dizziness, headache, weakness, gastrointestinal discomfort	[74]
Eleven doses of artesunate (6 mg/kg) given once every 15 days	94.48	Islet with embankment	Mild dizziness, headache, weakness, gastrointestinal discomfort	[74]
Eight doses of artesuante (6 mg/kg) given once every 7 days	100	Marshland region	2 cases with fever	[75]
Eleven doses of artesuante (6 mg/kg) given once every 15 days, with an additional dose 15 days post-exposure	100	Marshland region	2 cases with fever	[75]

In addition, the administration of artesunate at 900 mg first, followed by praziquantel at a daily dose of 40 mg/kg for three consecutive days was found to significantly improve the antischistosomal efficacy and alleviate the syndromes as compared to monotherapy [82,83]. However, we still need to devise optimal strategies to improve the preventive efficacy of artesunate-praziquantel combination against human *S. japonicum* infection.

### 3.3. Recommended Treatment Regimen

Currently, artesunate is recommended for prevention of *S. japonicum* human infection by multiple administration at 6 mg/kg starting from 7 days within the contact with infested water during the transmission season, with an additional treatment given 7 days post-exposure [43].

## 4. Dihydroartemisinin

Dihydroartemisinin, a derivative of artemisinin with the C-10 lactone group replaced by a hemiacetal, is the major active metabolite of artemisinin as well as of its derivatives, artemether and artesunate. This compound was developed in 1979 [84], and its antischistosomal activity was first discovered in 1980 [46]. It was found that oral administration with dihydroartemisinin at 150 and 300 mg/kg 7 days post-infection once a week for successive 4 weeks resulted in 76.1% and 89.1% worm burden reductions in *S. japonicum*-infected mice, and administration with dihydroartemisinin at 40 and 80 mg/kg 7 days post-infection once a week for successive 6 weeks reduced worm burdens by 99.2% and 100% in infected rabbits, while treatment with dihydroartemisinin at 45 mg/kg 7 days post-infection once a week for 4 successive weeks achieved a 98.7% worm burden reduction in infected dogs, respectively [85].

Recently, the *in vivo* activity of dihydroartemisinin was extensively evaluated in mice experimentally infected with *S. japonicum*. Administration with dihydroartemisinin at a single oral dose of 300 mg/kg 2 h, and 3, 5, 7, 10, 14, 18, 21, 28 or 35 days post-infection reduced total worm burdens of 1.1%, 15.2%, 31.3%, 64.8%, 22.0%, 32.7%, 18.1%, 14.5%, 48.5%, and 60.5%, respectively, indicating the high activity against 7-day schistosomula and 35-day mature schistosomes; however, no clear-cut dose-response relationship was observed in both the larval and adult stages [86]. An extended study to evaluate the efficacy of dihydroartemisinin administered at multiple doses or combined with praziquantel showed that the 3-day treatment with dihydroartemisinin at doses of 200, 300, 400 or 600 mg/kg on days 6–8 post-infection reduced total worm burdens of 69.2%, 80.7%, 87.1% and 90.6%, while the same treatment given on days 34–36 achieved total worm burden reductions of 83.8%, 92.9%, 94.1% and 95.3%, respectively; dihydroartemisinin-praziquantel combination administered at the juvenile stage did not appear more effective than treatment with dihydroartemisinin alone, while the combined treatment did not exhibit greater efficacy than that achieved by praziquantel treatment alone. In addition, treatment with dihydroartemisinin at multiple doses achieved no comparative efficacy against both juvenile and adult worms as compared to artemether or artesunate treatment at the same dose [87,88]. However, so far no human trials to evaluate the efficacy of dihydroartemisinin against schistosome have been reported. Therefore, further clinical trials to test the efficacy of dihydroartemisinin for the prevention and treatment of human *S. japonicum* infections, and investigate the underlying mechanisms of the antischistosomal action of dihydroartemisinin seem justified.

## 5. Conclusions

Experimental studies in animal models have demonstrated that the major artemisinin derivatives, including artemether, artesunate, and dihydroartemisinin, appear active against *S. japonicum*, particularly on schistosomulum [41,42], and the experiences from schistosomiasis control in China show that artemether and artesunate can play significant roles in the prevention of schistosomiasis japonica in China, particularly during the flood period when thousands of residents come in contact with schistosome-infested water [24,89]. Since there are no co-endemic areas for schistosomiasis japonica and falciparum malaria detected in China, there is no risk of drug resistance in *Plasmodium falciparum* in China, resulting from the use of artemisinin derivatives for the prevention and treatment of schistosomiasis japonica. Till now, the antischistosomal action of dihydroartemisinin has been evaluated in experimentally infected animal models, and the activity has not been validated in clinical trials. Therefore, we need to devise randomized, controlled trials to assess the efficacy and side effects of dihydroartemisinin for the treatment of human schistosome infections.

Although the antischistosomal actions of artemisinin derivatives have been identified for several decades, their exact mechanisms of action remain elusive till now. It has been suggested that artemether may interact with haemin and then cleave the endoperoxide bridge, and generate free radicals that may form a covalent bond with schistosome-specific proteins [90,91,92], and artemether was found to significantly decrease glycogen contents [93,94,95]. The currently available data demonstrate that artesunate treatment causes tegumental alternations and inhibition on energy metabolism of schistosomes [96,97]. In addition, it has been recently found that artesunate kills schistosome parasites through reducing the expression of schistosome glutathione reductase and cytochrome c peroxidase [98]. However, little is known on the mechanisms underlying antischistosomal actions of dihydroartemisinin, the major active metabolite of artemether and artesunate. The problem of unclear mechanisms of actions of these three artemisinin derivatives severely inhibits the development of other artemisinin analogues. Elucidation of the mechanisms underlying the antischistosomal activity of artemisinin derivatives by means of modern tools, such as –omics and RNA interference (RNAi), is therefore of urgent need. Currently, China is processing towards the elimination of schistosomiasis japonica [99]. However, the potential emergence of drug resistance would hamper the elimination of schistosomiasis japonica in China, since praziquantel is currently the only drug of choice for the treatment of human *S. japonicum* infections and praziquantel-based chemotherapy is still a major part of the national schistosomiasis control program implemented to control the morbidity and reduce the intensity of infection [30]. Fortunately, these three agents also appear mildly active against adult parasites [65]. In addition, recent investigations revealed no reduced sensitivity of artemether, artesunate and dihydroartemisinin in praziquantel-resistant *S. japonicum* [100,101]. These interesting findings promise to overcome the problem of praziquantel resistance, however, further studies are required to validate the hypothesis.

As old drugs identified firstly as antimalarials, artemisinin derivatives are creating new therapeutic possibilities. In addition to antimalarial and antischistosomal actions, they show activity against other parasites [102,103,104] and multiple cancers [105,106,107,108]. Artemisinin derivatives can hence be expected to open new perspectives for the treatment of additional diseases, besides malaria and schistosomiasis.

## References

[1] Colley D.G., Bustinduy A.L., Secor W.E., King C.H. (2014). Human schistosomiasis. Lancet.

[2] King C.H. (2010). Parasites and poverty: the case of schistosomiasis. Acta Trop..

[3] Zhou X.N., Bergquist R., Leonardo L., Yang G.J., Yang K., Sudomo M., Olveda R. (2010). Schistosomiasis japonica control and research needs. Adv. Parasitol..

[4] Chen M.G. (2014). Assessment of morbidity due to *Schistosoma japonicum* infection in China. Infect. Dis. Poverty.

[5] Dang H., Xu J., Li S.Z., Cao Z.G., Huang Y.X., Wu C.G., Tu Z.W., Zhou X.N. (2014). Monitoring the transmission of *Schistosoma japonicum* in potential risk regions of China, 2008–2012. Int. J. Environ. Res. Public Health.

[6] Hu Y., Xiong C., Zhang Z., Luo C., Ward M., Gao J., Zhang L., Jiang Q. (2014). Dynamics of spatial clustering of schistosomiasis in the Yangtze River Valley at the end of and following the World Bank Loan Project. Parasitol. Int..

[7] Li S.Z., Zheng H., Abe E.M., Yang K., Bergquist R., Qian Y.J., Zhang L.J., Xu Z.M., Xu J., Guo J.G. (2014). Reduction patterns of acute schistosomiasis in the People’s Republic of China. PLoS Negl. Trop. Dis..

[8] Zhou X.N., Guo J.G., Wu X.H., Jiang Q.W., Zheng J., Dang H., Wang X.H., Xu J., Zhu H.Q., Wu G.L. (2007). Epidemiology of schistosomiasis in the People’s Republic of China, 2004. Emerg. Infect. Dis..

[9] Doenhoff M.J., Cioli D., Utzinger J. (2008). Praziquantel: Mechanisms of action, resistance and new derivatives for schistosomiasis. Curr. Opin. Infect. Dis..

[10] Redman C.A., Robertson A., Fallon P.G., Modha J., Kusel J.R., Doenhoff M.J., Martin R.J. (1996). Praziquantel: An urgent and exciting challenge. Parasitol. Today.

[11] Cioli D., Pica-Mattoccia L. (2003). Praziquantel. Parasitol. Res..

[12] Wang W., Wang L., Liang Y.S. (2012). Susceptibility or resistance of praziquantel in human schistosomiasis: A review. Parasitol. Res..

[13] Fallon P.G. (1998). Schistosome resistance to praziquantel. Drug. Resist. Update.

[14] Doenhoff M.J., Kusel J.R., Coles G.C., Cioli D. (2002). Resistance of *Schistosoma mansoni* to praziquantel: Is there a problem?. Trans. R. Soc. Trop. Med. Hyg..

[15] Couto F.F., Coelho P.M., Araújo N., Kusel J.R., Katz N., Jannotti-Passos L.K., Mattos A.C. (2011). *Schistosoma mansoni*: A method for inducing resistance to praziquantel using infected *Biomphalaria glabrata* snails. Mem. Inst. Oswaldo Cruz.

[16] Fallon P.G., Doenhoff M.J. (1994). Drug-resistant schistosomiasis: Resistance to praziquantel and oxamniquine induced in *Schistosoma mansoni* in mice is drug specific. Am. J. Trop. Med. Hyg..

[17] Melman S.D., Steinauer M.L., Cunningham C., Kubatko L.S., Mwangi I.N., Wynn N.B., Mutuku M.W., Karanja D.M., Colley D.G., Black C.L. (2009). Reduced susceptibility to praziquantel among naturally occurring Kenyan isolates of *Schistosoma mansoni*. PLoS Negl. Trop. Dis..

[18] Ismail M., Botros S., Metwally A., William S., Farghally A., Tao L.F., Day T.A., Bennett J.L. (1999). Resistance to praziquantel: Direct evidence from *Schistosoma mansoni* isolated from Egyptian villagers. Am. J. Trop. Med. Hyg..

[19] Alonso D., Muñoz J., Gascón J., Valls M.E., Corachan M. (2006). Failure of standard treatment with praziquantel in two returned travelers with *Schistosoma haematobium* infection. Am. J. Trop. Med. Hyg..

[20] Silva I.M., Thiengo R., Conceição M.J., Rey L., Lenzi H.L., Pereira-Filho E., Ribeiro P.C. (2005). Therapeutic failure of praziquantel in the treatment of *Schistosoma haematobium* infection in Brazilians returning from Africa. Mem. Inst. Oswaldo Cruz.

[21] Silva I.M., Pereira-Filho E., Thiengo R., Ribeiro P.C., Conceição M.J., Panasco M., Lenzi H.L. (2008). Schistosomiasis haematobia: Histopathological course determined by cystoscopy in a patient in whom praziquantel treatment failed. Rev. Inst. Med. Trop. São Paulo.

[22] Chen M.G. (2005). Use of praziquantel for clinical treatment and morbidity control of schistosomiasis japonica in China: A review of 30 years’ experience. Acta Trop..

[23] Wu W., Huang Y. (2013). Application of praziquantel in schistosomiasis japonica control strategies in China. Parasitol. Res..

[24] Xiao S.H., Keiser J., Chen M.G., Tanner M., Utzinger J. (2010). Research and development of antischistosomal drugs in the People’s Republic of China a 60-year review. Adv. Parasitol..

[25] Wang W., Dai J.R., Li H.J., Shen X.H., Liang Y.S. (2012). The sensitivity of *Schistosoma japonicum* to praziquantel: A field evaluation in areas with low endemicity of China. Am. J. Trop. Med. Hyg..

[26] Wang W., Dai J.R., Li H.J., Shen X.H., Liang Y.S. (2010). Is there reduced susceptibility to praziquantel in *Schistosoma japonicum*? Evidence from China. Parasitology.

[27] Seto E.Y., Wong B.K., Lu D., Zhong B. (2011). Human schistosomiasis resistance to praziquantel in China: Should we be worried?. Am. J. Trop. Med. Hyg..

[28] Liang Y.S., Li H.J., Dai J.R., Wang W., Qu G.L., Tao Y.H., Xing Y.T., Li Y.Z., Qian K., Wei J.Y. (2011). Studies on resistance of *Schistosoma* to praziquantel XIII Resistance of *Schistosoma japonicum* to praziquantel is experimentally induced in laboratory. Chin. J. Schistosomiasis Control.

[29] Li H.J., Liang Y.S., Dai J.R., Wang W., Qu G.L., Li Y.Z., Xing Y.T., Tao Y.H., Qian K., Jia Y. (2011). Studies on resistance of *Schistosoma* to praziquantel XIV experimental comparison of susceptibility to praziquantel between PZQ-resistant isolates and PZQ-susceptible isolates of *Schistosoma japonicum* in stages of adult worms, miracidia and cercariae. Chin. J. Schistosomiasis Control.

[30] Zhou Y.B., Liang S., Jiang Q.W. (2012). Factors impacting on progress towards elimination of transmission of schistosomiasis japonica in China. Parasites Vector.

[31] Cioli D., Valle C., Angelucci F., Miele A.E. (2008). Will new antischistosomal drugs finally emerge?. Trends Parasitol..

[32] Miller L.H., Su X. (2011). Artemisinin: Discovery from the Chinese herbal garden. Cell.

[33] Tu Y.Y. (2011). The discovery of artemisinin (qinghaosu) and gifts from Chinese medicine. Nat. Med..

[34] Balint G.A. (2001). Artemisinin and its derivatives: An important new class of antimalarial agents. Pharmacol. Ther..

[35] Ansari M.T., Saify Z.S., Sultana N., Ahmad I., Saeed-Ul-Hassan S., Tariq I., Khanum M. (2013). Malaria and artemisinin derivatives: An updated review. Mini Rev. Med. Chem..

[36] Olliaro P.L., Taylor W.R. (2003). Antimalarial compounds: From bench to bedside. J. Exp. Biol..

[37] Nosten F., Hien T.T., White N.J. (1998). Use of artemisinin derivatives for the control of malaria. Med. Trop. (Mars).

[38] Haynes R.K. (2001). Artemisinin and derivatives: The future for malaria treatment?. Curr. Opin. Infect. Dis..

[39] Utzinger J., Xiao S.H., Tanner M., Keiser J. (2007). Artemisinins for schistosomiasis and beyond. Curr. Opin. Investig. Drugs.

[40] Keiser J., Utzinger J. (2012). Antimalarials in the treatment of schistosomiasis. Curr. Pharm. Des..

[41] Zhang X.G., Li G.X., Zhao S.S., Xu F.L., Wang Y.H., Wang W. (2014). A review of dihydroartemisinin as another gift from traditional Chinese medicine not only for malaria control but also for schistosomiasis control. Parasitol. Res..

[42] Liu R., Dong H.F., Jiang M.S. (2012). Artemisinin: The gifts from traditional Chinese medicine not only for malaria control but also for schistosomiasis control. Parasitol. Res..

[43] Liu R., Dong H.F., Guo Y., Zhao Q.P., Jiang M.S. (2011). Efficacy of praziquantel and artemisinin derivatives for the treatment and prevention of human schistosomiasis: A systematic review and meta-analysis. Parasites Vector.

[44] Wu W., Wang W., Huang Y.X. (2011). New insight into praziquantel against various developmental stages of schistosomes. Parasitol. Res..

[45] Pérez del Villar L., Burguillo F.J., López-Abán J., Muro A. (2012). Systematic review and meta-analysis of artemisinin based therapies for the treatment and prevention of schistosomiasis. PLoS One.

[46] Chen D.J., Fu L.F., Shao P.P., Wu F.Z., Fan C.Z., Shu H., Ren C.X., Sheng X.L. (1980). Experimental studies on antischistosomal activity of qinghaosu. Chin. Med. J..

[47] Le W.J., You J.Q., Mei J.Y., Wang G.F., Xie R.R. (1981). Antiscihstosomal action of some Qinghaosu derivatives in infected mice. Acta Pharmacol. Sin..

[48] Le W.J., You J.Q., Yang Y.Q., Mei J.Y., Guo H.F., Yang H.Z., Zhang X.W. (1982). Studies on the efficacy of artemether in experimental schistosomiasis. Acta Pharmacol. Sin..

[49] Xiao S.H., You J.Q., Yang Y.Q., Wang C.Z. (1995). Experimental studies on early treatment of schistosomal infection with artemether. Southeast Asian J. Trop. Med. Public Health.

[50] Xiao S., Shi Z., Zhuo S., Wang C., Zhang Z., Chu B., Zheng J., Chen M. (1996). Field studies on preventive effect of oral artemether against infection with schistosomal infection. Chin. Med. J. (Engl.).

[51] Xiao S.H., Wang J.L., Wang C.Z., Yang Z., Chu B., Yang H., Liu Y.H., Zheng J., Chen M.G. (1996). Protection of the residents from schistosome infection using oral artemether in mountainous endemic area. Chin. J. Parasitol. Parasit. Dis..

[52] Wang J.L., Xiao S.H., Yang Z., Huang M.H., Yang H., Liu Y.H., Zhou G.S., Zheng J., Chen M.G. (1997). Effect of oral artemether in controlling schistosomiasis in Yunnan mountainous endemic area. Chin. J. Parasitol. Parasit. Dis..

[53] Tian Z.Y., Xiao S.H., Xiao J.W., Liu D.S., Zhou Y.C., Zheng J., Chen M.G., Qu G.S., Zhang X.Y., Yao X.M. (1997). Reduction of *Schistosoma japonicum* infection in an endemic area in islet with embankment after prophylaxis with oral artemether throughout the transmission season. Chin. J. Parasitol. Parasit. Dis..

[54] Xu M.S., Xiao S.H., Song Q., Tao C.G., Xia C.G., Wang H., Chen M.G., Zheng J., Bu C.H., Hu F.Y. (1997). Observation on the effect of artemether on controlling schistosomiasis japonica in an endemic area of marshland. Chin. J. Parasitol. Parasit. Dis..

[55] Song Y., Xiao S., Wu W., Zhang S., Xie H., Xu X., Hu X., Cui Q., Chen M., Zheng J. (1998). Preventive effect of artemether on schistosome infection. Chin. Med. J. (Engl.).

[56] Li Y.S., Chen H.G., He H.B., Hou X.Y., Ellis M., McManus D.P. (2005). A double-blind field trial on the effects of artemether on *Schistosoma japonicum* infection in a highly endemic focus in southern China. Acta Trop..

[57] Chen H.G., Lin D.D., Li Y.S., Liu Y.M., McManus D.P., Huang X.H., Feng Z. (2006). Studies on effect of artemether to control infection and prevent acute infection of *Schistosoma japonicum* in high endemic areas. Chin. J. Schistosomiasis Control.

[58] Shuhua X., Jiqing Y., Jinying M., Huifang G., Peiying J., Tanner M. (2000). Effect of praziquantel together with artemether on *Schistosoma japonicum* parasites of different ages in rabbits. Parasitol. Int..

[59] Utzinger J., Chollet J., You J., Mei J., Tanner M., Xiao S. (2001). Effect of combined treatment with praziquantel and artemether on *Schistosoma japonicum* and *Schistosoma mansoni* in experimentally infected animals. Acta Trop..

[60] Hou X.Y., McManus D.P., Gray D.J., Balen J., Luo X.S., He Y.K., Ellis M., Williams G.M., Li Y.S. (2008). A randomized, double-blind, placebo-controlled trial of safety and efficacy of combined praziquantel and artemether treatment for acute schistosomiasis japonica in China. Bull. World Health Organ..

[61] Xiao S.H., Booth M., Tanner M. (2000). The prophylactic effects of artemether against *Schistosoma japonicum* infections. Parasitol. Today.

[62] Wu L.J., Xuan Y.X., Xu P.S., Li S.W. (1994). Study on artenibenzoate on prevention of schistosomiasis japonica. Chin. J. Schistosomiasis Control.

[63] Li S., Wu L., Liu Z., Hu L., Xu P., Xuan Y., Liu Y., Liu X., Fan J. (1996). Studies on prophylactic effect of artesunate on schistosomiasis japonica. Chin. Med. J. (Engl.).

[64] Wu L.J., Xu P.S., Fan J.T., Yang M.J., Li S.W. (1997). Experimental study on the prophylaxis of artesunate against acute schistosomiasis. Chin. J. Schistosomiasis Control.

[65] Ru W.W., Liang Y.S., Dai J.R., Li H.J., Xu Y.L., Zhu Y.C., Xu M. (2006). Studies on effect of artesunate against *Schistosoma japonicum* I Susceptibility of different developed stages of worms in mice to artesunate. Chin. J. Schistosomiasis Control.

[66] Wu L., Li S.W., Xuan Y.X., Xu P.S., Liu Z.D., Hu L.S., Zhou S.Y., Qiu Y.X., Liu Y.M. (1995). Field application of artesunate in prophylaxis of schistosomiasis: An observation of 346 cases. Chin. J. Schistosomiasis Control.

[67] Xu M.S., Zhu C.G., Wang H., Gao F.H., Wu Y.X., Cui D.Y., Zhang X.Z., Ou N., Wu Z.X. (1996). Study on preventive effect of artesunate against infection due to *Schistosoma japonicum* in an endemic area of marshlands. J. Trop. Dis. Parasitol..

[68] Liu Z.D., Hu F., Zhou S.Y., Liu Y.M., Hu L.S., Su L.H., Li S.W., Wu L.J. (1996). A clinical verification on artesunate for prevention of schistosomiasis japonica in Jishan. Chin. J. Schistosomiasis Control.

[69] Liu Z.D., Hu L.S., Liu Y.M., Hu G.H., Hu F., Qiu Y.X., Gao Z.L., Liu H.Y., Li J.Y., Su L.H. (1996). Expanded experimental study on the prevention of schistosomiasis japonica by oral artesunate. Chin. J. Parasit. Dis. Control.

[70] Xu M.S., Zhang S.Q., Wang T.P., Fang G.R., Wang Q.Z., He J.C., Zhang G.H., Chen J.R., Li J.T., Lu Y.S. (1998). Field application of oral artesunate for prevention of schistosomiasis japonica. J. Trop. Dis. Parasitol..

[71] Dai Y.H., Lv G.Y., Liu Z.Y., Zhang D.P. (1999). The effect of large-scale artesuante treatment on prevention of schistosomiasis during fighting a flood in the Yangtze River. Chin. J. Schistosomiasis Control.

[72] Lin D.D., Zhang S.J., Liu Y.M., Li S.W., Wu L.J., Gao Z.L., Tao B., Cheng Y.H. (1999). Field observation on the prophylaxis of artesuante with 15 days interval against infection of *Schistosoma japonicum*. Chin. J. Zoonoses.

[73] Liu H.Y., Liu Z.D., Hu L.S., Liu Y.M., Zhang S.J., Hu F., Cheng Y.H., Li S.W., Wu L.J., Xu P.S. (1999). Observation on the prevention of schistosomiasis japonica by long-term administration of artesunate. Chin. J. Parasit. Dis. Control.

[74] Lu G.Y., Lin G.J., Sun M.X., Cui J.F., Wu Q.Z., Li J.S. (2000). Study on scheme with oral artesunate for protecting people from infection of schistosome. Chin. J. Parasit. Dis. Control.

[75] Zhang SJ., Lin D.D., Liu Y.M., Liu H.Y., Liu Z.D., Hu L.S., Gao Z.L., Hu F., Xu M.S., Yi Z.H. (2000). Clinical trials on preventive effect of artesuante on schistosomiasis japonica. Mod. Diagn. Treat..

[76] Hua H.Y., Liang Y.S., Zhang Y., Wei J.F., Guo H.X. (2010). The sensitivity of artesunate against *Schistosoma japonicum* decreased after 10 years of use in China. Parasitol. Res..

[77] Liu R., Dong H., Jiang M.S. (2012). The sensitivity of artesunate against *Schistosoma japonicum* decreased after 10 years of use in China?. Parasitol. Res..

[78] Wu L.J., Xu P.S., Yang M.J., Fan J.T., Li S.W. (1998). Study on the interaction of artesunate and praziquantel in the treatment of schistosomiasis. Chin. J. Schistosomiasis Control.

[79] Zhang Y.P., Huang Y.X., Hong Q.B., Xu Y.L., Sun L.P., Xi W.P., Jiang Y.J., Wu F., Zhu Y.C. (2003). Observations on early co-treatment with artesunate and praziquantel for schistosomiasis in rabbits. Chin. J. Schistosomiasis Control.

[80] Zhang Y.P., Huang Y.X., Liang Y.S., Xu Y.L., Yang K., Hong Q.B., Jiang Y.J., Xi W.P., Zhu Y.C. (2005). Effect of combined therapy of artesunate and praziquantel for different intensity of *Schistosoma japonicum* infection in rabbits. Chin. J. Parasit. Dis. Control.

[81] Xia C.S., Zhang X.Y., Liu Y.F., Jiang Z.H., Li N.P. (2000). Observation on preventive effect of artesunate plus praziquantel on schistosomiasis japonica. J. Prev. Med. Chin. PLA.

[82] Li Y.F. (2006). The combined treatment of artesunate and praziquantel on acute schistosomiasis. J. Med. Pest. Control.

[83] Wang S.R., Wu X.F., Zhang Z.H., Zhang J.M., Zhou Z.W., Wang D.G. (2007). Acute schistosomiasis treatment with artesunate and praziquantel: A report of 38 cases. Chin. J. Schistosomiasis Control.

[84] Li Y., Yu P.L., Chen Y.X., Li L.Q., Gai Y.Z., Wang D.S., Zheng Y.P. (1979). Synthesis of some derivatives of artemisinine. Chin. Sci. Bull..

[85] Li S.W., Wu L.J., Yi Y.X., Xu P.S. (1988). Primary experiment of artemisinin derivatives in prevention of schistosomiasis. Sichuan J. Physiol. Sci..

[86] Li H.J., Wang W., Qu G.L., Tao Y.H., Xing Y.T., Li Y.Z., Wei J.Y., Dai J.R., Liang Y.S. (2011). *In vivo* activity of dihydroartemisinin against *Schistosoma japonicum*. Ann. Trop. Med. Parasitol..

[87] Li H.J., Wang W., Tao Y.H., Qu G.L., Xing Y.T., Li Y.Z., Wei J.Y., Dai J.R., Liang Y.S. (2011). Dihydroartemisinin-praziquantel combinations and multiple doses of dihydroartemisinin in the treatment of *Schistosoma japonicum* in experimentally infected mice. Ann. Trop. Med. Parasitol..

[88] Li H.J., Wang W., Li Y.Z., Qu G.L., Xing Y.T., Tao Y.H., Wei J.Y., Dai J.R., Liang Y.S. (2011). Effects of artemether, artesunate and dihydroartemisinin administered orally at multiple doses or combination in treatment of mice infected with *Schistosoma japonicum*. Parasitol. Res..

[89] Xiao S.H. (2005). Development of antischistosomal drugs in China, with particular consideration to praziquantel and the artemisinins. Acta Trop..

[90] Xiao S., Chollet J., Utzinger J., Matile H., Mei J., Tanner M. (2001). Artemether administered together with haemin damages schistosomes *in vitro*. Trans. R. Soc. Trop. Med. Hyg..

[91] Xiao S.H., Wu Y.L., Tanner M., Wu W.M., Utzinger J., Mei J.Y., Scorneaux B., Chollet J., Zhai Z. (2003). *Schistosoma japonicum*: *In vitro* effects of artemether combined with haemin depend on cultivation media and appraisal of artemether products appearing in the media. Parasitol. Res..

[92] Xiao S.H., You J.Q., Gao H.F., Mei J.Y., Jiao P.Y., Chollet J., Tanner M., Utzinger J. (2002). *Schistosoma japonicum*: Effect of artemether on glutathione S-transferase and superoxide dismutase. Exp. Parasitol..

[93] Shuhua X., Hotez P.J., Tanner M. (2000). Artemether, an effective new agent for chemoprophylaxis against shistosomiasis in China: Its *in vivo* effect on the biochemical metabolism of the Asian schistosome. Southeast Asian J. Trop. Med. Public Health.

[94] Xiao S.H., You J.Q., Mei J.Y., Guo H.F., Jiao P.Y., Sun H.L., Yao M.Y., Feng Z. (1997). Effect of artemether on glucose uptake and glycogen content in *Schistosoma japonicum*. Acta Pharmacol. Sin..

[95] You J., Guo H., Mei J., Jiao P., Feng J., Yao M., Xiao S. (1994). Effect of artemether on glycogen, protein, alkaline phosphatase and acid phosphatase of *Schistosoma japonicum*. Chin. J. Parasitol. Parasit. Dis..

[96] Wu L.J., Xu Y.X., Guo Y., Xu P.S., Li S.W. (1996). Studies on the effect of artesunate to the energy-metabolic enzymes of *Schistosoma japonicum*. Chin. J. Schistosomiasis Control.

[97] Guo Y., Xu P.S., Xuan Y.X., Wu L.J., Li S.W. (1997). Effect of artesunate on ultrastructure of schistosomula *Schistosoma japonicum*. Chin. J. Schistosomiasis Control.

[98] Ashour D.S., Shoheib Z.S., Abdeen A.A. (2012). Artesunate effect on schistosome thioredoxin glutathione reductase and cytochrome c peroxidase as new molecular targets in *Schistosoma mansoni*-infected mice. Parasitol. United J..

[99] Rollinson D., Knopp S., Levitz S., Stothard J.R., Tchuem Tchuenté L.A., Garba A., Mohammed K.A., Schur N., Person B., Colley D.G. (2013). Time to set the agenda for schistosomiasis elimination. Acta Trop..

[100] Wang W., Li H.J., Qu G.L., Xing Y.T., Yang Z.K., Dai J.R., Liang Y.S. (2014). Is there a reduced sensitivity of dihydroartemisinin against praziquantel-resistant *Schistosoma japonicum*?. Parasitol. Res..

[101] Wang W., Li T.Y., Ji Y., Qu G.L., Qian Y.L., Li H.J., Dai J.R., Liang Y.S. (2014). Efficacy of artemether and artesunate in mice infected with praziquantel non-susceptible isolate of *Schistosoma japonicum*. Parasitol. Res..

[102] Ru W.W., Liang Y.S. (2006). Progress of research on artemisinin against parasitic diseases. Chin. J. Schistosomiasis Control.

[103] Li H.J., Wang W., Liang Y.S. (2011). Advances in research of dihydroartemisinin against parasitic diseases. Chin. J. Schistosomiasis Control.

[104] Ho W.E., Peh H.Y., Chan T.K., Wong W.S. (2014). Artemisinins: Pharmacological actions beyond anti-malarial. Pharmacol. Ther..

[105] Chaturvedi D., Goswami A., Saikia P.P., Barua N.C., Rao P.G. (2010). Artemisinin and its derivatives: A novel class of anti-malarial and anti-cancer agents. Chem. Soc. Rev..

[106] Firestone G.L., Sundar S.N. (2009). Anticancer activities of artemisinin and its bioactive derivatives. Expert Rev. Mol. Med..

[107] Nakase I., Lai H., Singh N.P., Sasaki T. (2008). Anticancer properties of artemisinin derivatives and their targeted delivery by transferrin conjugation. Int. J. Pharm..

[108] Singh N.P., Lai H.C. (2004). Artemisinin induces apoptosis in human cancer cells. Anticancer Res..

